# Data quality evaluation in wearable monitoring

**DOI:** 10.1038/s41598-022-25949-x

**Published:** 2022-12-10

**Authors:** Sebastian Böttcher, Solveig Vieluf, Elisa Bruno, Boney Joseph, Nino Epitashvili, Andrea Biondi, Nicolas Zabler, Martin Glasstetter, Matthias Dümpelmann, Kristof Van Laerhoven, Mona Nasseri, Benjamin H. Brinkman, Mark P. Richardson, Andreas Schulze-Bonhage, Tobias Loddenkemper

**Affiliations:** 1 Department of Neurosurgery, Epilepsy Center, Medical Center – University of Freiburg, Freiburg, Germany; 2 Ubiquitous Computing, Department of Electrical Engineering and Computer Science, University of Siegen, Siegen, Germany; 3 Division of Epilepsy and Clinical Neurophysiology, Boston Children’s Hospital, Harvard Medical School, Boston, MS USA; 4 Department of Basic and Clinical Neuroscience, Institute of Psychiatry, Psychology and Neuroscience, King’s College, London, UK; 5 Bioelectronics Neurophysiology and Engineering Laboratory, Department of Neurology, Mayo Clinic, Rochester, MN USA; 6 Department of Microsystems Engineering (IMTEK), University of Freiburg, Freiburg, Germany; 7 School of Engineering, University of North Florida, Jacksonville, FL USA

**Keywords:** Biomedical engineering, Epilepsy

## Abstract

Wearable recordings of neurophysiological signals captured from the wrist offer enormous potential for seizure monitoring. Yet, data quality remains one of the most challenging factors that impact data reliability. We suggest a combined data quality assessment tool for the evaluation of multimodal wearable data. We analyzed data from patients with epilepsy from four epilepsy centers. Patients wore wristbands recording accelerometry, electrodermal activity, blood volume pulse, and skin temperature. We calculated data completeness and assessed the time the device was worn (on-body), and modality-specific signal quality scores. We included 37,166 h from 632 patients in the inpatient and 90,776 h from 39 patients in the outpatient setting. All modalities were affected by artifacts. Data loss was higher when using data streaming (up to 49% among inpatient cohorts, averaged across respective recordings) as compared to onboard device recording and storage (up to 9%). On-body scores, estimating the percentage of time a device was worn on the body, were consistently high across cohorts (more than 80%). Signal quality of some modalities, based on established indices, was higher at night than during the day. A uniformly reported data quality and multimodal signal quality index is feasible, makes study results more comparable, and contributes to the development of devices and evaluation routines necessary for seizure monitoring.

## Introduction

Ambulatory seizure monitoring using wearables is now within reach^1–7^, but continuous data collection in outpatient, real-life situations and data analysis in real-time bear multiple challenges before this can be effectively implemented. In particular, established tools on how to determine the data quality of wearable signals as a basis for data selection are currently missing.

The quality of the raw recorded data from wearables is a frequently underreported aspect in clinical studies employing these wearables, especially in quantitative terms. Furthermore, the quality of data can be expressed in many different ways, and data quality measures may also depend on the aims of the related analysis and project^8–13^. Additionally, data quality is decisive for data accuracy and reliability^14–16^. Seizure monitoring, compared to many other clinical applications, requires a high temporal resolution, as seizures can be as short as a few seconds^17–19^. In this context, knowledge of artifacts and tools to assess data quality and generate data reliability ratings are crucial for subsequent analysis and consequently outcome reliability. Artifacts can occur specific to a single modality or across multiple different modalities, such that the separate and simultaneous consideration of the different modalities may contribute to the assessment of data quality.

Our overarching goal was to assess data quality in wearables, using the Empatica E4 in the setting of epilepsy monitoring as a specific example. Our main hypothesis was that signal quality affects the recording of neurophysiological modalities’ activity from the wrist, and in case of major disruptions may influence overall recording quality. Having a baseline comparison between data sets of different sites and in different settings may considerably improve the interpretability of results in other studies using similar devices. We aimed to describe and identify common artifacts that impair recording quality. Additionally, we aimed to develop and evaluate a tool for data quality processing in wearables and implement this tool at four epilepsy centers. Here, we specifically define the term *data quality* as the overarching description of metrics used to quantitatively determine the goodness of data in terms of data completeness (percentage of recorded vs. expected samples, i.e., the device was off or not recording for some reason), on-body score (percentage of time estimated as worn on the body, as opposed to recording while placed elsewhere), as well as individual modality-specific scoring. These monomodal signal metrics are in turn referred to as *signal quality* throughout.

## Methods

### Exemplary data and signal artifact overview

As a first step in creating a wearable data quality assessment tool, we purposefully recorded data from two healthy and consenting volunteers. The first recording consisted of multiple 30-s long periods testing different external artifact sources. The intent was to create a small reference data set that we can use to comprehensively visualize artifacts depending on their sources and validate our data quality measures. We recorded sections with the device in its normal state at rest, during random motion, and during repetitive wrist rotation and translation. Furthermore, we tested the device in several simulated real-life scenarios, including partially or completely off the body and with light shining directly on it. In a second test recording, we collected data during a multi-hour period of typical daily living. With this recording, we were able to validate the data quality measures in an at-home context. We also used this recording to visualize inherent changes in data quality over a representative day, like changes between wakefulness and sleep. Both data sets helped in designing and fine-tuning the on-body scoring and signal quality metrics, and were subsequently visualized with these scores highlighted.

### Overview of inpatient data collection procedures

Additionally, we collected wearable data through independent protocols at four international epilepsy centers: Boston Children’s Hospital (BCH), King’s College London (KCL), Mayo Clinic Rochester (MCR), and Medical Center University of Freiburg (Universitätsklinikum Freiburg, UKF). Since data were collected independently, there were variations in our patient enrollment and data collection procedures between our cohorts. For this study, assessing data quality in a variety of settings and across centers, we provide a brief overview of center-specific data collection processes and common data aspects applicable to this article for inpatient (Table 1) and outpatient (Table 2) data sets. For additional in-depth information on these recording procedures with wearable devices beyond this outline, please also refer to previous publications^20–23^.

**Table 1:** Overview of inpatient EMU cohorts and recruitment.

	BCH	KCL	MCR	UKF
Study	Detect, predict and prevent seizures	RADAR-CNS	MySeizureGauge	RADAR-CNS
EC/IRB approval	IRB-P00001945	16/LO/2209	18-008357	538/16
Enrollment period	Feb. 2015–Feb. 2021	Jun. 2017–Aug. 2019	Nov. 2018–Dec. 2019	Jul. 2017–Mar. 2020
# of patients	415	29	20	172
Recruitment age range	0–29	18–80	3–70	7–80
Actual age range (median, 95% CI)	9.35 [0.8, 20.7]	38.0 [20.4, 63.8]	21.5 [11.1, 54.9]	30.0 [14.7, 64.0]
Sex (% female)	49.4%	48.3%	30.0%	47.1%
Exclusion	Sensitive skin or history of skin condition Allergic to rubber or materials like rubber Warning of severe aggressive behavior Sensory disorders or sensitivity to objects touching the skin Electrical implants, such as pacemakers or VNS Acutely ill or in distress	Established diagnosis of psychogenic non-epileptic attacks as the only seizure type Frequent vigorous involuntary movements or frequent parasomnias with major motor components Inability to comply with the trial procedure, such as cognitive or behavioral problems	Cognitive or psychiatric conditions render patients unable to cooperate with data collection, or manage and recharge devices Presence of open or healing wounds near monitoring sites	Established diagnosis of psychogenic non-epileptic attacks as the only seizure type Frequent vigorous involuntary movements or frequent parasomnias with major motor components Inability to comply with the trial procedure, such as cognitive or behavioral problems
Wearable placement	Wrist/ankle	Wrist	Wrist	Wrist
Recording mode	Device	Streaming	Device	Streaming

*EC* Ethics Committee, *IRB* Institutional Review Board, *LTM* long-time monitoring, *EMU* epilepsy monitoring unit.

**Table 2:** Overview of outpatient ambulatory cohorts and recruitment.

	KCL	MCR	UKF
Study	RADAR-CNS	MySeizureGauge	RADAR-CNS
EC/IRB approval	19/LO/1884	18-008357	605/19
Enrollment period	Feb. 2021–Dec. 2021	Dec. 2019–Feb. 2022	Jan. 2021–Nov. 2021
Follow up	Up to 6 months	Up to 12 months	Up to 6 months
# of patients	15	14	12
Recruitment age range	18–70	3–70	18–70
Actual age range (median, 95% CI)	36.0 [33.6, 45.6]	39.5 [30.7, 44.2]	29.5 [27.5, 42.3]
Sex (% female)	40.0%	64.3%	50.0%
Exclusion	Established diagnosis of psychogenic non-epileptic attacks as the only seizure type Frequent vigorous involuntary movements or frequent parasomnias with major motor components Inability to comply with the trial procedure, such as cognitive or behavioral problems Unwillingness to use an Android smartphone	Cognitive or psychiatric conditions render patients unable to cooperate with data collection, or manage and recharge devices Presence of open or healing wounds near monitoring sites	Established diagnosis of psychogenic non-epileptic attacks as the only seizure type Frequent vigorous involuntary movements or frequent parasomnias with major motor components Inability to comply with the trial procedure, such as cognitive or behavioral problems Unwillingness to use an Android smartphone
Wearable placement	Wrist	Wrist	Wrist
Recording mode	Streaming	Device	Streaming

*EC* Ethics Committee, *IRB* Institutional Review Board.

We enrolled patients at all centers within prospective cohort studies in the inpatient setting, and at three centers in the outpatient setting. All inpatients underwent clinically indicated video-electroencephalography (video-EEG) monitoring in the epilepsy monitoring unit. All enrolled patients wore an E4 biosensor (Empatica, Milan, Italy).

Written Informed Consent was obtained from all patients or their legal guardians. The study at BCH was approved by the Boston Children’s Hospital Institutional Review Board under ID number IRB-P00001945. The studies at KCL were approved by the London Fulham Research Ethics Committee under ID numbers 16/LO/2209 (inpatient) and 19/LO/1884 (outpatient). The studies at MCR were approved by the Mayo Clinic Institutional Review Board under ID number 18-008357 (inpatient and outpatient). The studies at UKF were approved by the Ethics Committee at the University of Freiburg under ID numbers 538/16 (inpatient) and 605/19 (outpatient). All research was performed in accordance with the relevant guidelines and regulations.

### Overview of outpatient data collection procedures

KCL, MCR, and UKF also conducted ambulatory studies in addition to the in-hospital ones, which have slightly diverging study procedures to account for the uncontrolled and ultra-long-term recordings (Table 2).

### Sensor and recorded signals

The Empatica E4 (Empatica Inc, Boston, MA, USA) is a research-grade wearable device capable of recording accelerometry (ACC), electrodermal activity (EDA), photoplethysmography (PPG), and skin temperature (TEMP) signals^24^. It was chosen over other consumer-grade wearable devices and fitness trackers because it is specifically rated for epilepsy monitoring, and has been successfully used in other epilepsy-focused studies and beyond. Further, the E4 provides raw data from multiple biosignal sensors at sample rates meaningful for seizure detection. The device has a Conformité Européenne (CE) class 2a certification as a medical device. It can be worn around the wrist or ankle, and can record data either locally to the device or stream it directly via Bluetooth, to a smartphone app for example. In the case of local data recording, the data must then be downloaded via a computer and USB cable once the recording is done or the internal memory of the device is full. The battery life of the device ranges from 24 h (streaming mode) to 48 h (device memory mode). Technical specifications of the Empatica E4 wearable device are listed in Table 3.

**Table 3:** Overview of technical specifications of the Empatica E4 device.

	Empatica E4
Manufacturer	Empatica Inc, Boston, MA, USA
Certification	CE class IIa
Body position	Wrist, ankle
Biosignals	ACC, EDA, PPG, TEMP
Sampling rates	32 Hz, 4 Hz, 64 Hz, 4 Hz
ACC range	± 2 g
Battery life	24-48 h
Recording mode	Device, streaming

Photoplethysmography (PPG) is an optical measurement method to determine changes in volume in the blood flow of a specific body part (blood volume pulse, BVP). The shape of a clean PPG signal is closely related to the blood pulse wave of the human body^25^ (see Fig. 2a, BVP zoomed during rest condition). The heart rate is thereby directly derivable from the peak to peak intervals between single systolic peaks. The raw output signal of a PPG sensor is a function of the amount of reflected light falling into the photoelectric sensor.

Temperature (TEMP) is recorded from the skin and does not reflect the core body temperature. The sensor can thus also capture qualitative changes induced by ambient temperature.

Sweat production changes the electric properties of the skin which can be captured by electrodermal activity (EDA) recordings. Two components that contribute to measurable changes in EDA have been described. The fast galvanic skin responses, also referred to as phasic responses, act in the order of 0.5 to 5 s, whereas the tonic component, which expresses in level changes, acts in the order of minutes^26,27^.

### Recordings and device settings

We recorded data at BCH and MCR in device memory mode and transferred it from the device after the recording period. The data was uploaded from the device to the Empatica cloud and the files were downloaded for offline analysis. Data was manually synchronized to the local video-EEG system. Recordings included here were filtered by duration. We included only those with at least 1 h of data for the inpatient cohorts and 24 h of data for the outpatient cohorts.

At KCL and UKF the device was regularly swapped with a fully charged one twice per day and was running in streaming mode for the entire duration of the recording, connected to a base device with Android OS and a recording app independent of the device manufacturer^28^. Raw data and timestamps of the wearable device and the Android device were stored for later analysis. For the in-hospital study, wearable data was synchronized with the video-EEG system and stored on-premises. For the ambulatory study, data was automatically transferred over the internet to a remote server, and regular data completeness reports were generated to monitor for data loss.

### Data quality metrics toolkit

#### Data completeness assessment

**Figure 1:**
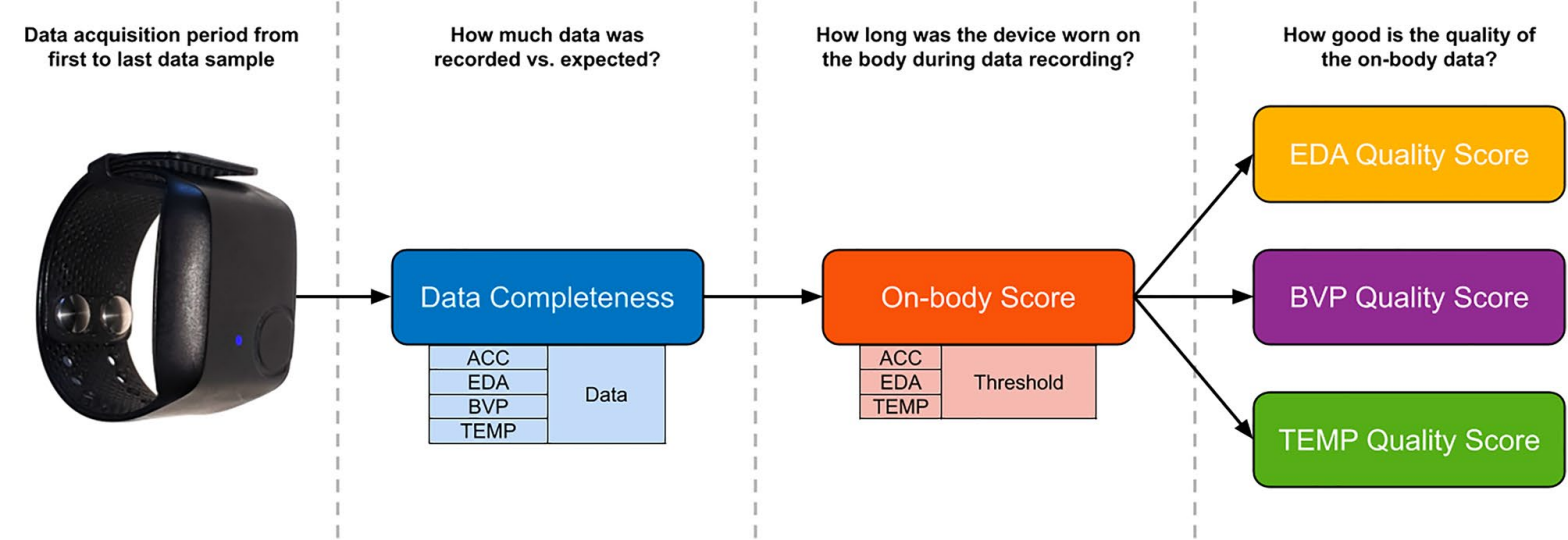
Data quality assessment pipeline. The amount of data referenced in each step decreases from left to right, that is, the data completeness score is relative to the whole recording period, whereas the individual signal quality scores are relative to the amount of data that was estimated as on-body.

We gauged the functionality of the device and the compliance of study participants by calculating data completeness scores. Here, data completeness and its inverse data loss are strictly defined as the presence and absence of data samples, e.g., due to the device having been turned off or otherwise not recording during the enrollment period of a subject, regardless of the quality of the data which is assessed in the other metrics. To this end, we computed the total duration of the data set from the number of recorded samples and the recording sample rate. To obtain the completeness scores, we then calculated the ratio of recorded duration and expected duration, i.e., the time difference between the first and last sample. Figure 1 gives a simplified overview of the complete data quality analysis pipeline. To account for minor differences in relative numbers of recorded samples per modality, we used the maximum over all modalities for the recording duration and the average for the completeness score. Thus, the completeness (arb. unit) and duration (seconds) of the data sets were calculated based on the following equations:

$$Completeness = mean\left( {\frac{{N_{rec} }}{{F_{s} \cdot \left( {t_{end} - t_{start} } \right)}}} \right),$$

$$Duration = max\left( {\frac{{N_{rec} }}{{F_{s} }}} \right),$$

whereby $N_{rec}$ is the number of recorded samples per modality, $F_{s}$ is the sample rate per modality, and $t_{start}$ and $t_{end}$ are the first and last data samples in a modality recording, respectively.

#### Device on-body check

As the device may not always be on the body, we developed an on-body assessment metric estimating the percentage of time the device was actually worn properly on the body, as opposed to recording while placed elsewhere. This metric does not include deficits due to data loss, which are already covered in the data completeness score. To achieve this, a combination of metrics can be analyzed from available biosignal modalities:

##### ACC

Assuming that the subject did not wear the device, the level of activity in a certain window can be thresholded, and if it is below a certain level of activity for a specific percentage of the data points within the window, the on-body status for that window is 0, otherwise it is 1. Here, we set this value as 0.2 of the 10-s moving standard deviation^29^. The level of activity was calculated as the moving standard deviation in a 10-s window of the per-sample sum over the three ACC axes.

##### EDA

The EDA signal falls to a zero value once the electrodes are not in contact with the skin anymore. The raw signal can simply be thresholded over a window, and if it is below some level of activity for a certain percentage of the data points in the window, the on-body status for that window is 0, otherwise, it is 1. Here, we set this threshold value as 0.05 μS^9,30^.

##### TEMP

The temperature values while the device is worn should fall within some reasonable values for skin or body temperature. Thus, if the temperature falls outside of this range for a certain percentage of the data points in the window, the on-body status for that window is 0, otherwise, it is 1. Here, we set this temperature range between 25 and 40 °C^12,31^.

##### On-body metric calculation

The BVP modality was not used in the on-body assessment because its signal is heavily influenced by external light and thus has a random and undefined range and shape during off-body segments, depending on factors of the incoming light like intensity or color. We set the window length over which the on-body status is evaluated to 1 min. The minimum percentage of time points per window needed within the respective on-body ranges was 1%. The three scores can be used independently or in combination, depending on the desired sensitivity of the check. Thus, each 1-min window will have a single score, 0 or 1, to denote whether the device is deemed off-body or on-body during that window, respectively. For the results presented here, we set a threshold of at least one modality showing as on-body, for the entire segment to be considered on-body. The remaining signal quality assessment described below is performed on segments of the data where the device was on-body.

#### Signal quality assessment and scoring

We screened all data recorded from the four inpatient and three outpatient cohorts with established quality metrics. For ACC the on-body score serves as a quality indicator and we did not calculate additional separate data quality scores for the ACC signal. We developed modality-specific quality metrics for TEMP, EDA, and BVP. For these signals, we determined signal quality checks and scored per 1-min data interval to calculate the ratio of intervals that passed the quality check, estimating the overall data quality.

The signal quality scoring for both electrodermal activity and temperature was very similar, due to the sources and sensors being closely related, due to calculations based on range thresholds. For EDA, we set the range of limited signal quality below a signal amplitude of 0.05 μS^9,30,32^. Specifically, this value delimits zero lines, i.e., loss of contact, from actual data values. For the TEMP signal, we set the range of valid values from 25 to 40 °C^12,31^. As the temperature value represents at most the skin temperature rather than the body temperature, a slightly lower band of reasonable temperatures than might be expected was chosen to demarcate meaningful values.

The rate of amplitude change (RAC) here is calculated as the ratio of the difference between the highest and lowest value in the window, and either the maximum or minimum value, whichever comes first. In addition to the threshold checks, the RAC was calculated in two-second windows for the respective signals, independently^9,32^. As both the EDA and TEMP signals usually should not contain high-frequency changes in their normal characteristics (even including phasic EDA events), the RAC was thus thresholded to be below a factor of 0.2 for the signal to be considered valuable^9,32^. That is, if the signal showed a > 20% increase or decrease within a 2-s window, that window was considered of bad quality. We then combined the results from both the thresholding and the RAC method by logical conjunction, such that both tests needed to pass for a sample to be counted as good quality.

For the BVP signal, we utilized spectral entropy to assess the signal quality. A clean BVP signal from the Empatica E4 without noise from motion artifacts has a smooth quasi-periodic signature, which can be separated from random noise or, more importantly, from motion artifacts by analyzing the entropy of the signal spectrum. We calculated this index in 4-s windows, with overlap and linear interpolation, and for a frequency band of 0.1 to 5 Hz^8,9,23,33^. The metric values range between 0 for a signal with a single spectral component, that is, a perfectly periodic signal, and 1 for a signal with a constant spectrum, that is, perfectly random noise. We empirically determined the threshold above which the raw BVP input signal would be considered of poor quality as 0.8^9^ for this study. Like for the EDA and TEMP signals, this thus gives a binary quality score for each data sample of the BVP signal.

To generate more meaningful signal quality results and to improve the feasibility of plotting results, each signal quality index result was further processed by averaging over a 60-s window, providing an individual score for each of the modalities for each minute of data in the range of 0 to 1, with 1 denoting the best possible signal quality. We then averaged scores for the entire data set of a single participant, and subsequently for all participants in a cohort.

To analyze differences concerning specific characteristics like recording location and time of day, we implemented filters to divide the data sets into distinct groups. For the BCH data set, every recording had a specific location attribute, either ‘wrist’ or ‘ankle’. To analyze the effect of recording location, the score aggregation was done on each of these groups separately. Furthermore, to examine signal quality changes during day- and nighttime, we filtered the minute-wise scores by their timestamps. Scores between 8 am and 8 pm were grouped as daytime scores, and nighttime scores between 8 pm and 8 am. Thereby we also took into account the four different time zones where the centers were located.

### Statistical analysis

To evaluate differences in data and signal quality scores for their statistical significance we use the two-tailed two-sample t-test statistic and report the t-value, the degrees of freedom, and the p-value in Supplement 1. All computations of data and signal quality analysis as well as the statistical analysis of the results were done using MATLAB R2022a (MathWorks, Natick, MA, USA).

## Results

### Characteristics of typical artifacts in wearable data

**Figure 2:**
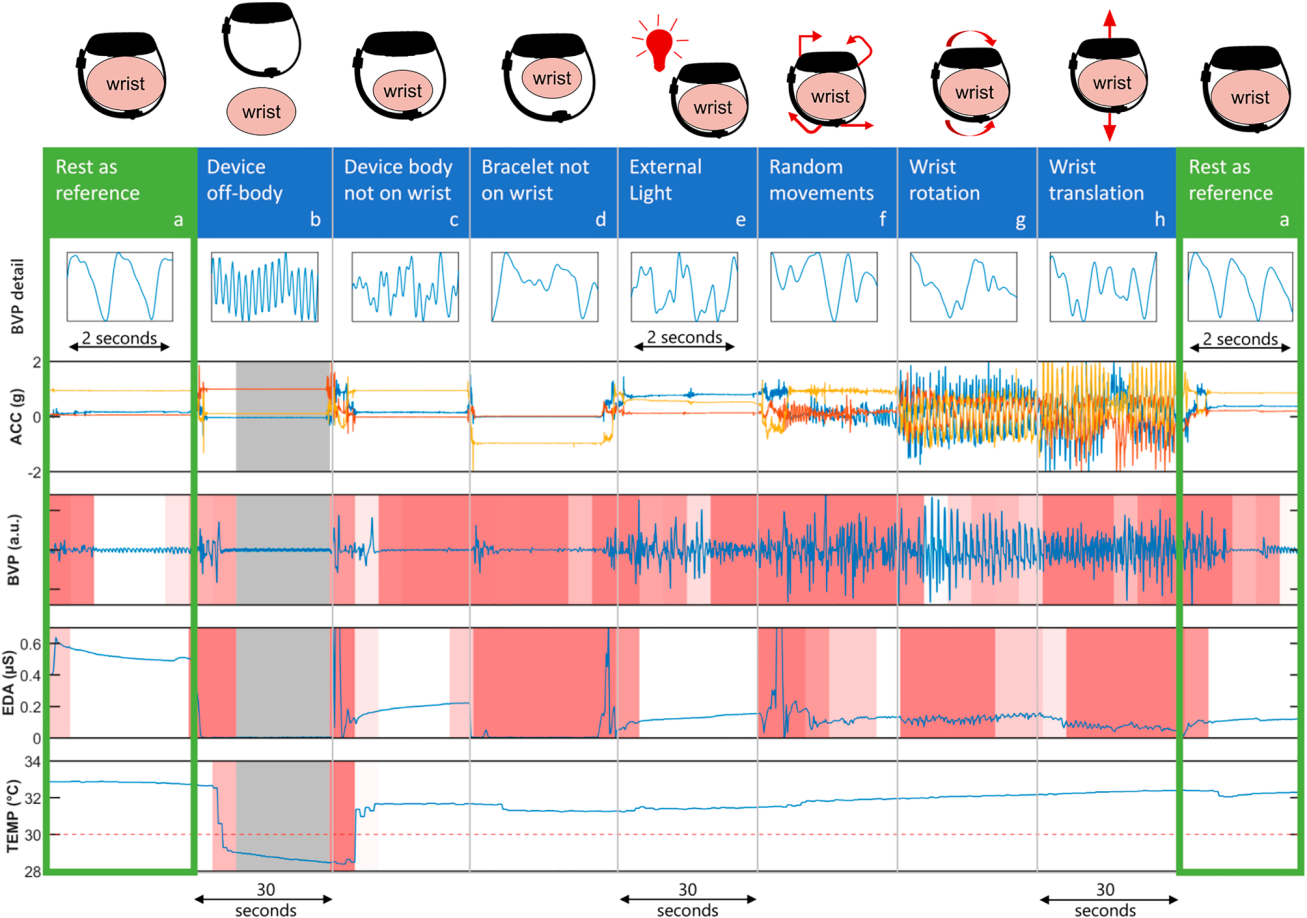
Visualization of typical artifacts in E4 data, with on-body and signal quality scores marked. Plots from top to bottom: BVP detail, ACC (x, y, z), BVP, EDA, TEMP. Off-body periods are highlighted in gray. Periods of low EDA, BVP, and TEMP signal quality scores are highlighted in red (the lighter the color the better the quality, no color = 100% quality). (**a**) Reference recording during rest, blood pulse wave without artifacts, no motion, baseline EDA and TEMP. (**b**) Wristband not on the person, BVP shows a noise-like pattern, no motion is recorded, and EDA and TEMP drop to 0 or room temperature, respectively. Note, that TEMP changes might show a delay. (**c**) Device body is not on the wrist, BVP cannot be recorded well as the sensor loses skin contact. (**d**) Wristband is not on the wrist, the EDA drops to 0 as the integrated sensor electrodes lose contact. (**e**) External light shining on the device and into the sensor can greatly affect the photosensor’s (BVP) recordings. (**f**) Random movements, (**g**) wrist rotation, and (**h**) wrist translation (e.g., moving up and down) disrupt BVP, ACC, and EDA signals, causing rhythmic changes and peaks to show across modalities.

Typical artifacts in wearable data of the Empatica E4 sensor, recording accelerometry (ACC), electrodermal activity (EDA), blood volume pulse (BVP), and skin temperature (TEMP), are depicted in Fig. 2. We selected seven frequently occurring artifacts that affect the signal quality and the information content of the signal differently and illustrated them in comparison to a resting measurement. The effect of artifacts on the signals ranges from no meaningful information being recorded when the wristband is not on the patient to differential and minor effects on data completeness and consistency.

**Figure 3:**
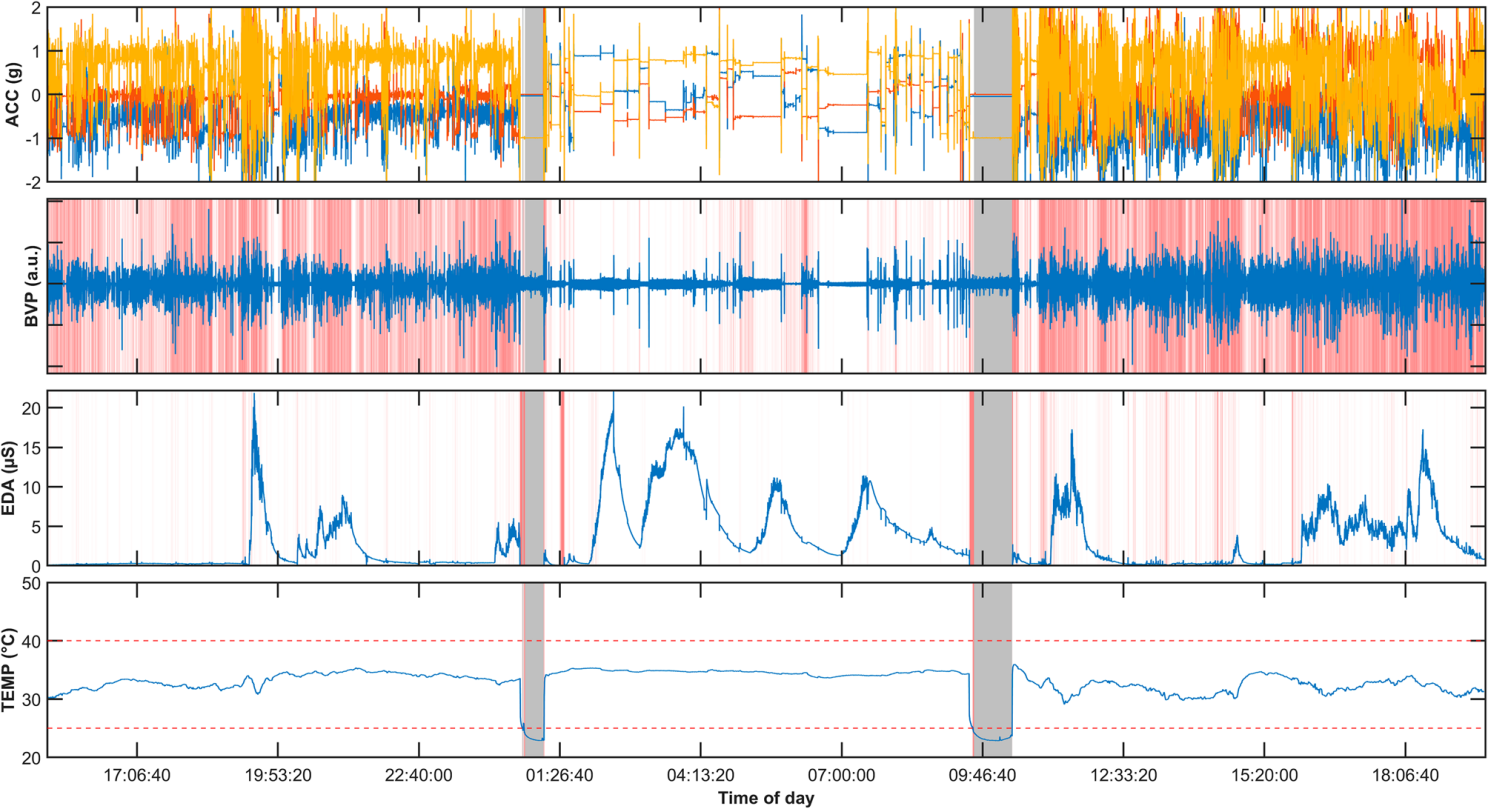
Example of ~ 24 h of long-term ambulatory data from the Empatica E4 device (healthy test subject). Plots from top to bottom: ACC (x, y, z), BVP, EDA, TEMP. Off-body periods are highlighted in gray. Periods of low EDA and BVP signal quality scores are highlighted in red (the lighter the color the better the quality, no color = 100% quality). The recording is divided into three parts by the two off-body periods. Left: first day afternoon and evening; Middle: night; Right: morning and rest of the second day.

Visual analysis of E4 data recorded during a typical long-term period of daily living permits distinguishing intervals of wakefulness and sleep based on the raw ACC and BVP data and the BVP signal quality scores, based on overall less movement and variability during sleep. Furthermore, off-body time periods are characterized by a temperature drop in TEMP, random noise or regular oscillations in BVP, and a low amplitude in ACC variance and EDA (Fig. 3).

### Patient cohorts

**Figure 4:**
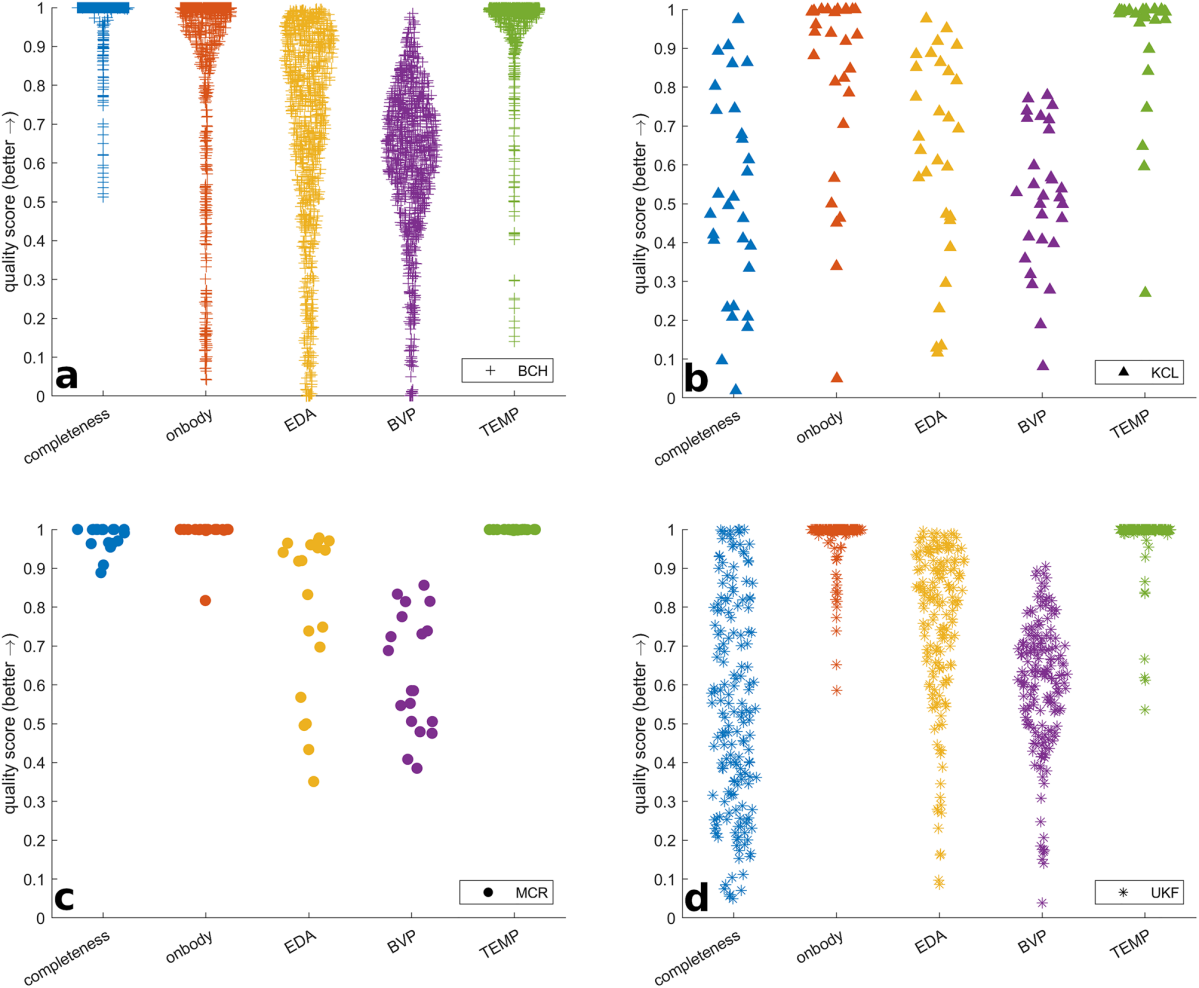
Swarm plots of data quality score distributions for the inpatient cohorts of (**a**) BCH (N = 832), (**b**) KCL (N = 29), (**c**) MCR (N = 19), and (**d**) UKF (N = 169).

**Figure 5:**
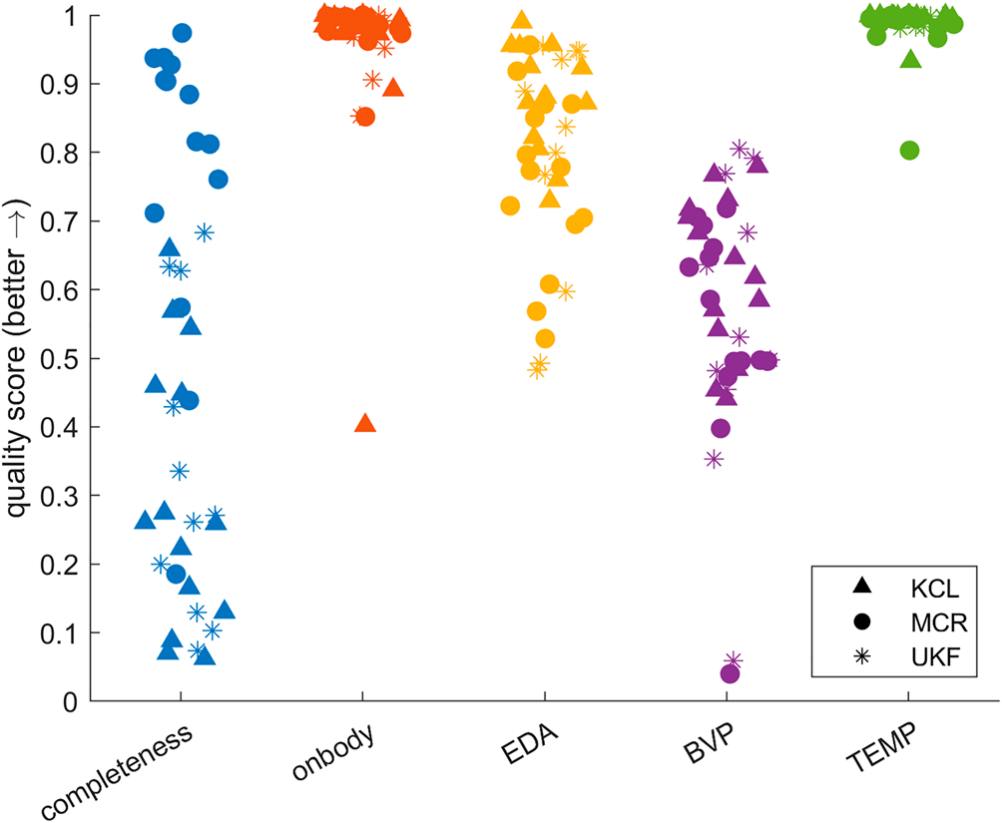
Swarm plot of the data quality score distributions for the outpatient cohorts (N = 39).

We included 37,166 h from 632 patients recorded in the inpatient setting and 90,776 h from 39 patients recorded in the outpatient setting. See the “Methods” section for more details on the four centers where data was recorded, and the respective cohorts. In the Boston Children’s Hospital (BCH) inpatient dataset, for some patients, multiple recordings were available, including recordings during multiple admissions and parallel recordings, for example, with one E4 on a wrist and one on an ankle. For children with small wrists the ankle placement is an alternative. Else, the placement was determined based on the tolerability of the patient and in consultation with the care team. Thus, a total of 832 recordings were obtained from 415 individual patients. In the ambulatory datasets from the King’s College London (KCL) and Medical Center University of Freiburg (Universitätsklinikum Freiburg, UKF) sites, several participants dropped out of the study before they completed the planned 6-month follow-up (KCL N = 5/15; UKF N = 1/12). Furthermore, for some patients, the follow-up period was deliberately shortened to 3 months (KCL N = 3/15; UKF N = 1/12). These data were included in this quality assessment. Tables 4 and 5 present the aggregated scores for all participants of the respective cohorts, i.e., the mean, median, standard deviation, minimum value, and maximum value. Furthermore, Figs. 4 and 5 further visualize these results in swarm plots per participant, for the inpatient and outpatient cohorts, respectively.

**Table 4:** Data quality results for the inpatient cohort data, presented as the aggregated scores for all participants of the respective cohort.

BCH	N patients = 415*					Sum h:m:s = 25,600:30:56
	Completeness (%)	On-body (%)	EDA^†^ (%)	BVP^†^ (%)	TEMP^†^ (%)	Duration h:m:s
Mean	98.4	88.3	68.9	60.6	95.5	30:46:11
Median	100.0	99.7	75.9	63.3	99.4	23:49:25
Std	6.4	23.0	26.6	19.7	12.2	16:54:30
Min	51.3	4.2	0.0	0.0	7.5	01:15:44
Max	100.0	100.0	99.6	98.5	100.0	96:29:16

KCL	N patients = 29					Sum h:m:s = 2181:43:27
	Completeness (%)	On-body (%)	EDA^†^ (%)	BVP^†^ (%)	TEMP^†^ (%)	Duration h:m:s
Mean	51.5	82.4	62.7	51.5	92.4	75:13:54
Median	49.6	93.9	67.2	52.0	99.3	49:22:04
Std	26.5	24.8	26.2	18.1	16.4	63:40:27
Min	1.9	5.0	11.6	8.1	27.0	04:04:48
Max	97.5	100.0	97.6	77.9	100.0	284:04:31

MCR	N patients = 19					Sum h:m:s = 1825:33:38
	Completeness (%)	On-body (%)	EDA^†^ (%)	BVP^†^ (%)	TEMP^†^ (%)	Duration h:m:s
Mean	97.9	99.0	78.4	63.2	99.9	96:04:55
Median	100.0	100.0	91.9	58.5	100.0	89:56:13
Std	3.3	4.2	21.4	15.3	0.1	49:03:13
Min	88.9	81.7	35.1	38.5	99.7	19:59:16
Max	100.0	100.0	97.8	85.6	100.0	224:23:33

UKF	N patients = 169					Sum h:m:s = 7557:47:47
	Completeness (%)	On-body (%)	EDA^†^ (%)	BVP^†^ (%)	TEMP^†^ (%)	Duration h:m:s
Mean	54.2	98.0	75.7	60.0	98.6	44:43:14
Median	52.7	100.0	81.8	62.4	100.0	36:30:10
Std	27.0	6.0	20.4	16.5	6.3	36:56:26
Min	5.0	58.6	8.6	3.9	53.6	01:17:34
Max	100.0	100.0	99.6	90.5	100.0	243:27:47

We report the amount of data recorded between the start and end of the recording as completeness. From this, we test how long the device is worn on the body, and score the signal quality of the modalities EDA, BVP, and TEMP. Additionally, we report the duration of the recorded data in h:m:s = hours:minutes:seconds.

*N recordings = 832, some patients had multiple admissions and multiple simultaneous devices.

^†^Signal quality scores are in relation to data periods estimated as on-body in the previous step.

**Table 5:** Data quality results for the outpatient cohort data, presented as the aggregated scores for all participants of the respective cohort.

KCL	N patients = 14*					Sum h:m:s = 11,209:23:59
	Completeness (%)	On-body (%)	EDA^†^ (%)	BVP^†^ (%)	TEMP^†^ (%)	Duration h:m:s
Mean	30.1	93.9	88.0	62.3	99.3	800:40:17
Median	26.0	98.4	88.1	63.2	99.9	683:50:17
Std	20.0	15.7	7.8	11.4	1.8	799:28:26
Min	6.3	40.2	72.9	44.1	93.3	57:07:04
Max	65.8	100.0	99.0	77.9	99.9	2977:36:48

MCR	N patients = 14					Sum h:m:s = 65,394:52:36
	Completeness (%)	On-body (%)	EDA^†^ (%)	BVP^†^ (%)	TEMP^†^ (%)	Duration h:m:s
Mean	76.9	97.8	76.0	53.9	97.6	4671:03:45
Median	85.0	99.0	77.6	54.1	99.3	4933:49:38
Std	22.7	3.8	13.0	17.6	5.1	2663:24:57
Min	18.5	85.2	52.9	4.0	80.3	270:09:58
Max	97.4	100.0	95.6	71.8	100.0	8097:26:03

UKF	N patients = 11*					Sum h:m:s = 14,172:02:42
	Completeness (%)	On-body (%)	EDA^†^ (%)	BVP^†^ (%)	TEMP^†^ (%)	Duration h:m:s
Mean	34.1	96.5	78.7	55.1	99.3	1288:22:03
Median	27.1	98.6	83.7	53.1	99.7	1137:18:34
Std	22.3	4.6	18.2	22.2	0.7	939:58:26
Min	7.3	85.3	48.3	6.0	98.1	312:01:18
Max	68.3	100.0	95.6	80.5	100.0	3000:34:58

We report the amount of data recorded between start and end of the recording as completeness. From this we test how long the device is worn on the body, and score the signal quality of the modalities EDA, BVP, and TEMP. Additionally we report the duration of the recorded data in h:m:s = hours:minutes:seconds.

*Includes data from dropped-out participants, and those with deliberately shortened follow-up.

^†^Signal quality scores are in relation to data periods estimated as on-body in the previous step.

### Results of completeness, on-body, and signal quality tools

We used multimodal data quality metrics (data completeness, on-body score) and modality-specific signal quality metrics (EDA, BVP, TEMP) to assess data loss, compliance, and amount of data containing artifacts, in a structured and quantitative manner over multiple independent cohorts. We used these metrics to compare inpatient and outpatient data quality, investigate whether recording location makes a difference, and evaluate the effect of time of day on the recording quality. Tables 4 and 5 summarize the results of the data and signal quality analysis per cohort and per metric. Statistical values are presented in Supplement 1.

#### Data completeness

Results show a difference between cohorts using the device in memory recording mode and cohorts using the data streaming mode (p < 0.001). While data loss (i.e., the inverse of data completeness) in the former datasets was consistently below 10%, the latter had up to 50% loss on average, with variances across individual recordings. Accordingly, the range between the minimum and the maximum data completeness for streamed recordings is much higher, ranging from almost no data recorded to all possible data recorded.

#### On-body score

On-body scores were consistently high across all cohorts, with average values above 80%. While some outliers in some cohorts exist, the variance among individuals was also relatively low, suggesting overall good compliance regarding wearing the device as uninterrupted as possible.

#### Modality-specific signal quality

For all cohorts, the TEMP data had the highest signal quality (mean = 96.1%), followed by EDA (mean = 70.4%) and BVP (mean = 60.2%). BVP also consistently had the lowest maximum quality per cohort.

#### Comparison of inpatient and outpatient results

Comparing the in-hospital (Fig. 4) and ambulatory (Fig. 5) data sets over the three centers that recorded both revealed no differences in data or signal quality measures. However, follow-up analysis per site revealed that for individual sites inpatient data showed higher data completeness values and for one site EDA signal quality was better than in the outpatient setting (see Supplement 1 for p-values).

#### Comparison of different device placements

In the BCH inpatient cohort, participants wore devices on the wrist (N = 383) or the ankle (N = 447). Recording location did not affect the data completeness and on-body scores; in both scenarios, the mean scores differed by only 0.1%. For the BVP and TEMP modalities, the average signal quality scores at the wrist were lower by 4.7% (p < 0.001) and 2.1% (p = 0.015), respectively.

#### Comparison of the day- and nighttime recordings

**Figure 6:**
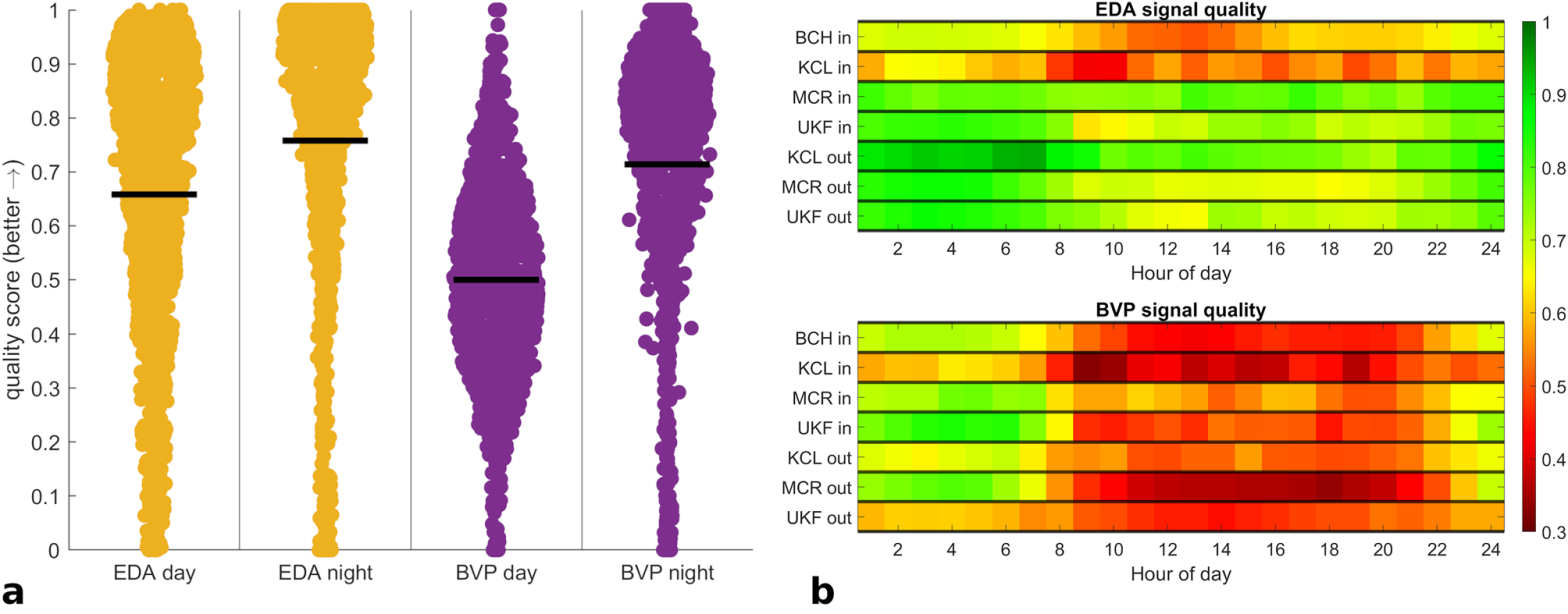
Comparison of EDA and BVP signal quality scores when grouping data by day- and nighttime.

A diurnal cycle is identifiable when visually inspecting data and quality scores over longer periods (Fig. 3). We confirm this in our recorded data sets by filtering the data quality results grouped by time of day (Fig. 6), separately aggregating scores during daytime (8 am to 8 pm) and nighttime (8 pm to 8 am). BVP and EDA indicate a difference between day- and nighttime scores (both p < 0.001). Over all cohorts, the mean of BVP scores is 49.9% during the daytime, but 70.7% at night. EDA scores at night (mean = 75.1%) are higher than day scores (mean 65.6%). The data completeness during the night (88.5%) is also better than during the day (82.8%; p < 0.001). The on-body scores and TEMP signal quality did not differ between day- and nighttime recordings (both < 1% mean difference).

(**a**) Data from all seven cohorts is shown together (N = 1094). Horizontal bars mark the overall mean, both differences are statistically significant (p < 0.001). (**b)** Average signal quality by the hour of the day and by cohort.

## Discussion

Ambulatory recordings using wearable devices are gradually becoming part of the diagnostic toolbox for monitoring patients with diseases of the central nervous system. Completeness and quality aspects are critical for practical implementation, especially in the field of epilepsy where seizures can happen on a scale of seconds to a few minutes. Each sample of data can potentially contain relevant information, and signal quality must be assessed thoroughly. Signal quality assessments have been done under more general circumstances^10,34–37^, but wearable data quality in the context of epilepsy has not been a widely researched topic so far^8,9,38^.

We created a qualitative visualization of wearable artifacts and developed a tool for the quantitative analysis of data quality based on wearable data from seven cohorts of epilepsy patients. Artifact sources are multifaceted (Fig. 2). Motion artifacts are a primary cause of bad signal quality, and research has suggested various methods for mitigating these^26,39,40^. Overall, we found that temperature measurements are least impacted by artifacts, followed by EDA and BVP recordings. The recording mode of the device had the highest impact on data loss (Table 4, Fig. 4). Moving to an ambulatory setting may further amplify the impact on data loss. However, our data set also showed slightly higher quality for EDA signals in this setting (Table 5, Fig. 5). We found that the recording location, here wrist or ankle, and the time of day can impact the signal quality for all modalities, especially for BVP and EDA. Visual inspection of data and quality scores over extended periods (Fig. 3) reveals the influence of time of day, seeing that a diurnal cycle is identifiable.

### Different artifacts have different multimodal signatures

The signal quality of wearable data is affected by a multitude of artifacts (Figs. 2, 3). Artifacts originate from the environment, the person wearing the wearable, and the device itself^41^. Device-specific artifacts are often difficult to detect and typically include electrical noise, indirect changes in the signal due to a rise in the operating temperature of the device over time, time shifts, and calibration offsets^20^. These types of artifacts are not further evaluated in this study. Person-specific artifacts comprise motion artifacts and improperly worn devices, which can both lead to sensors losing skin contact, as well as accidental power off. Artifacts can affect modalities differently. While a complete loss of contact results in a 0-line for the EDA signal, motion artifacts without contact loss result in abrupt level changes or high amplitude fluctuations, and PPG shows considerable deviation from expected signal patterns^24,42^.

Ambient artifacts are for example light artifacts interfering with the light of the PPG sensor^43^, weather conditions, such as high humidity which increases the moisture level of the skin surface and thus its conductivity^44^, as well as coverage of the device for example by a blanket which increases the temperature and thereby potentially EDA levels^45^. The temperature and EDA levels are also likely to be influenced heavily by the season when the recording takes place^46^. Ambient temperatures inside as well as outside may differ based on season. The temperature thresholds defined for the on-body and signal quality assessment in this study were set with standard indoor temperatures in mind and could be adjusted for seasonal changes in future studies. Furthermore, assessing only relative changes in the data or employing normalization techniques may be advisable.

Other major factors potentially impacting the recording quality for wearable devices are the location on the body the device is attached to, and the time of day the recording took place, both of which were further analyzed in this study and are discussed below. In addition, there are some other aspects that may influence data quality which we did not further evaluate in this study. Prominently, the skin color of the subject has been a major point of discussion concerning the quality of PPG recordings^14,47,48^. As the sensor directly depends on reflectance properties of the skin tissue, different skin colors may change the overall signal quality of the recorded PPG data^49,50^. In this context, the Empatica E4 device has been reported in one study to perform worse than some consumer-grade wearable devices^14^. Nevertheless, these other devices are not certified as medical devices for epilepsy monitoring, and generally do not provide direct access to raw sensor data like the Empatica E4 does. In the studies presented here we did not focus on the aspect of skin color and further analysis is needed in future work. Moreover, the overall design of the device can affect patient comfort and thereby compliance^51^, and patients in whom seizures are difficult to detect due to atypical tracings in EDA recordings have been reported in related work^52,53^.

### Data completeness and on-body score depend on recording setting and are comparable across different centers

The recording mode of the wearable device was the main cause of differences in data loss, with the two centers using the streaming mode showing considerably less overall data completeness (Table 4). The device memory mode is very reliable and data loss only occurs whenever the device is switched off to swap it or when using the shower, as seen in previous studies^20,21,54^. But, this comes with considerable additional effort for the user, who needs to regularly connect the device to a computer to synchronize the data and free up device memory, as well as additional data privacy considerations introduced by the device manufacturer’s cloud service. Conversely, the streaming mode allows for direct and uninterrupted control of the data, at the expense of data completeness, losing on average about half of the data in the cohorts presented here (Table 4), likely due to Bluetooth range constraints^20^.

Furthermore, in our studies across centers utilizing the device, the battery life tends to worsen with prolonged and regular use^55^ in the range of only a few months^20^. We also found that the sampling rate has a certain drift over time, such that timestamps for data samples can be inaccurate by up to one second per hour of recording^20^. These issues may also have had some minor effects on the data completeness calculations.

Any longer-term data sets from wearable devices, especially if recorded in ambulatory settings, will contain periods where the device was recording, but not actually on the subject’s body in the correct manner, as also noted in previous studies^21,30^. This could, for example, happen due to the subject removing the device for a short amount of time to do activities like washing, heavy-duty work, or sports, while leaving the device on and recording. Since the data from such periods contains no relevant information, data may need to be edited accordingly before performing the signal quality checks suggested here. Moreover, some research has investigated avoiding this problem altogether by further developing the devices, e.g., integrating wearables with the human body beyond wristbands or similar devices^56^.

Another study assessed the performance of patients with epilepsy in self-managing wearable devices^38^. Their questionnaire-based results are gathered from a cohort wearing the same device used in this study, operated in streaming mode. Their cohort is a subset of the KCL inpatient cohort presented here. Study participants had the best compliance concerning wearing the device and correctly fitting it, while compliance was worst when it came to pairing the device to a Bluetooth companion device, with frequent connection issues. These results directly coincide with our assessment of the data in terms of good on-body scores and bad data completeness.

### This multicenter study confirms and extends the findings and methods of previous studies on modality-specific signal quality

BVP data is dependent on measuring reflected light from a light-emitting diode (LED), and this signal can be heavily skewed by contamination from external light sources and motion artifacts, sometimes to the point of being irrecoverable through retrospective data processing^40,57,58^. Thus, any movement of the device and, by extension, the body part it is attached to, may result in motion artifacts in the signal. Direct sunlight falling on the wearable could also have a considerable effect on the data quality. These conditions have a direct impact on BVP data recorded over multiple days, which is reflected by the overall worst signal quality results in our cohorts for this modality. In another study, the feasibility of using the BVP signal to detect epileptic seizures was analyzed considering peri-ictal periods only^8^. Thus, these results are not directly comparable to ours which are based on entire data sets including inter-ictal periods. A different study group also investigated the signal quality of BVP data recorded from patients with epilepsy^9^. They however do not provide a quantitative statement that would be comparable to our results. Still, our signal quality metric is building on, expanding, and validating this research group’s average values for what they describe as good, marginal, and noise data. Moreover, one study reports 94% overall good quality in their estimated heart rate based on PPG data, but their data set is restricted to recordings at night and they do not further specify the signal quality measure they apply^59^.

Wearables record thermal activity by measuring the skin temperature at the recording location. This is often lower than commonly known values for human core body temperature, e.g., when determining fever, and is more prone to environmental influences^60^. Like other responses of the sympathetic nervous system, changes in peripheral body temperature can take multiple seconds and might occur delayed. While thermoregulation in the context of epilepsy has been a topic of some research in the past^61,62^, its causes, effects, and interactions are not well understood. Furthermore, when the sensor is covered for some time, the environmental temperature may increase, which could result in a higher temperature recorded from the wrist. As part of thermoregulation, the patient might then also sweat more due to the increased environmental temperature and EDA might increase. Overall, the relevance of skin temperature measurements for epilepsy monitoring awaits further exploration.

Besides thermoregulatory processes, other responses of the sympathetic nervous system like piloerection or psychophysiological arousal may also induce changes in the EDA signal^44^. EDA is typically recorded by dry electrodes which do not necessarily need to be adhesive, as long as they have continuous contact with the skin, as is the case with the Empatica E4 device used here. Artifacts in the EDA signal are thus often caused by connection loss of the electrodes to the skin due to, e.g., body motion, and present as a sharp decrease or increase, or fall to a zero-line value altogether^12^. These kinds of artifacts can be detected by simple thresholding as well as analysis of the rate of amplitude change^9,12^, similar to the artifacts seen in the temperature signal. The circumstantial and short-term nature of these artifacts is also reflected in our results by the highest overall variances per modality. Still, a majority of individual recordings score higher than 75% adequate EDA data. Our study builds on an EDA signal quality analysis, using the same device and presenting similar metrics applied to data^9^. These authors report an average of 35.7% marginal or noise segments per minute of EDA data. In our terms, this would directly compare to an EDA signal quality score of 64.3%, congruent with the results shown here of EDA scores between 50 and 80%.

Accelerometry is captured by a sensor measuring activity induced by motion. The design and functionality of ACC sensors may lead to sensor noise and other inconsistencies like sensor saturation or displacement^63–65^. However, these sensors do not produce measurable artifacts with any practical relevance to the topic at hand^13^. Any movement large enough to cause a change in the sensor could be measured, and there are no external, physical, or other reasons for the sensor to produce a change in the signal if there was no movement. Thus, we did not evaluate the data quality of the ACC signal in more detail.

In seizure monitoring, well-established wearables are the Empatica Embrace and E4^66^. The E4 is a research device that allows accessing raw data and, in addition to the sensors integrated into the Embrace device, records cardiac activity. Therefore, all centers used the E4. The Empatica E4 device, specifically, has been used in a number of other studies related to epilepsy monitoring as well as in other contexts. Naturally, the signal quality of PPG data in general is often discussed in other work as it is highly susceptible to motion artifacts^57^, however, there is also a high variance in PPG signal quality across different devices^67^, such that a device-specific consideration seems appropriate. One study, for example, reports that more than half of epileptic seizures in a data set could not be detected from Empatica E4 PPG data due to motion artifacts^68^. In another study assessing PPG as a measure for epileptic seizures, the data from three out of eleven patients wearing an Empatica E4 device was not usable, since no seizure periods without motion artifacts could be recorded^69^. The device has also been evaluated in general purpose real-world settings, with results indicating that the Empatica E4 may be unsuited for monitoring everyday activities^70,71^. Even so, it remains the only certified research-grade epilepsy monitoring device on the market that provides raw data for all of the modalities investigated here, relevant to epileptic seizure detection. The Empatica EmbracePlus is an upcoming wearable device featuring the same signal modalities as the E4, but at this time no studies using the device have been published yet, and a data and signal quality review similar to this one will be necessary. Moreover, the E4 has been shown to facilitate seizure detection by heart rate estimation from PPG despite its susceptibility to motion artifacts^8,72^.

### Large amounts of data can be collected in the outpatient setting, but reduced control is reflected in less data completeness

In ultra-long-term monitoring, patients are in a much less controlled environment and recordings can last for multiple months, instead of multiple days as is the case for inpatient recordings under more controlled conditions. Therefore, biosignal recordings may be of overall lesser quality^12^. In our data, however, ambulatory recordings did not necessarily indicate worse performance in terms of data quality. Data completeness was not significantly different in the outpatient setting when comparing overall cohorts, but differences were significant for individual centers, as would be expected considering the largely unsupervised data collection procedures. However, the outpatient datasets all have comparably small cohort sizes such that the results may be skewed. On-body scores were comparably high in all cohorts, although the smaller cohorts and more targeted recruitment process could have introduced some selection bias with regard to compliance. Comparing signal quality for individual modalities showed that BVP data have the lowest quality for in-hospital and outpatient data, underlining its high susceptibility to measurement artifacts^57^. Interestingly, the ambulatory results do not significantly differ from the inpatient results for any of the signal modalities, suggesting that the presumably more frequently occurring motion artifacts in the outpatient setting did not have a significant effect on signal quality. This may be explained by the smaller and more selective ambulatory cohorts.

### Ankle placement offers an additional recording location for pediatric patients

We also investigated if different recording locations on the body have any influence on data quality. At the BCH site, where predominantly pediatric patients were recruited, participants wore the device at either the ankle or the wrist. While there was no substantial difference in quality for data completeness and on-body scores, the recording location did seem to have some effect on the quality of the recorded signals, as has been suggested in some other studies^26,58,73,74^. BVP and TEMP signal quality was better when the wristband was placed on the ankle. This could be explained by the relatively fewer movements of the ankle location as opposed to the wrist, especially in the inpatient setting. Lower EDA quality at the ankle is likely due to sweat gland distributions and exact sensor placement on the ankle, i.e., medial or lateral. Patients or caregivers choose the ankle location based on comfort and where other medical devices are placed. Furthermore, the ankle placement might be more unobtrusive in the outpatient setting. Concerning epilepsy monitoring, the placement of the wearable device on the ankle could provide an additional opportunity for better recordings in pediatric patients.

### Wearables offer a good option for nighttime monitoring

Overall data quality is higher during nighttime compared to daytime. This finding in our large cohort is in line with findings in smaller samples specifically for PPG data^35,75^. Higher data completeness during the night likely results from fewer risks for the wearable device to be disconnected, such as moving away from the recording device in the outpatient setting, or otherwise stopping recording accidentally. The differences in EDA and BVP signal quality may be related to reduced movement during sleep, as well as usually darker environments specifically concerning the BVP sensor. Thus, this divergence might be even more pronounced if a standard 8-h night or sleep estimation, or an individual sleep detection, were applied to the comparison. In the context of epilepsy monitoring, this is particularly relevant for various reasons. Epileptic seizures are underreported in manual seizure diaries, especially during the night^76^. Furthermore, the risk of sudden unexpected death in epilepsy patients is higher during the night^77^. As such, robust wearable monitoring systems would substantially improve both seizure reporting as well as alarms during the nighttime periods in patients’ daily living.

### Limitations

Results need to be interpreted in the setting of data collection. While some key aspects of the cohorts from the four international centers align, like the choice of the wearable device and the study participant inclusion criteria, the results also need to be interpreted in the setting of minor variations in the data acquisition setups. The participant recruitment and enrollment procedures were not consistently aligned between the centers. There also were technical differences between the devices used in the studies. While all recordings were performed with an Empatica E4 device, different hardware and firmware versions may have been used across and even within cohorts. Furthermore, besides a rough range of up to 14 days for inpatient recordings and up to 12 months for outpatient recordings, the target recording length was undefined in all cohorts. Additionally, beyond differences in demographics and device placements, deployment in different regions may have contributed to variability^78^. Enrollment procedures for the ambulatory studies also partially included new patients who did not necessarily participate in an inpatient study. The selection process for some cohorts targeted patients with a high perceived potential for compliance, based on familiarity with wearable technology and willingness to participate in months-long studies.

Our cohorts are furthermore subject to selection and information bias. Pediatric inpatients were mostly drug-refractory and therefore results might not be generalizable to other patients, however, the impact on data quality is presumably small. Similarly, we did not take medication effects into account, while some anti-seizure medications might influence autonomic activities. Especially in the outpatient settings, we could not control for environmental temperature that impacts TEMP and EDA measures. Our current separation of the day- and nighttime recordings does not reflect sleep times. While in the inpatient setting sleep time relates to hospital routines, we had no way of determining sleep times in outpatient recordings. Therefore we decided to split the day into halves to make comparisons easier. In addition, we did not analyze in detail any differences in data and signal quality with respect to age, particularly concerning very young patients recruited in the BCH cohort.

Lastly, while the BVP signal quality measure applied in this study has been used in some other research, it has not been extensively validated concerning its meaningfulness in robustly estimating a heart rate. While there is cursory evidence that it correctly maps periods of good and poor signal quality to estimations of the heart rate^8,9^, a thorough validation is still planned in the future. This validation will need to include a new data collection protocol under laboratory conditions with alternative ground truth recordings for all signal modalities. Especially for EDA and TEMP recordings, no currently accepted gold standard exists beyond the methods employed here^79^, and would need to be established in this further validation. Nevertheless, the analysis of sample artifact and ambulatory data we conducted in this study (see Figs. 2, 3) suggests a good accordance of the metrics with actual signal quality and on-body state of the device. Overall, reproducibility of results across different cohorts and in different settings suggests an overall robust data acquisition paradigm.

## Conclusion

We present an approach to assessing data quality from wearable recordings and apply our methodology to data sets recorded across four international epilepsy centers in the inpatient and outpatient settings. We provide a detailed overview of typical artifacts influencing wrist-worn non-EEG wearable data collection and implement a comprehensive tool to appraise this data in terms of data completeness, on-body score, and signal quality. Signal recording quality affects all autonomic modalities, and especially blood volume pulse recordings. Artifact recognition and data quality ratings may provide additional value and improve precision, may serve as a standard metric in experimental studies, and foster further design improvements for future wearable device studies in epilepsy research. While all of the signal quality metrics employed in this study have been used in other research before, some still lack a structured and comprehensive validation on ground truth data, which is a necessary next step. Remote monitoring is feasible for patients with epilepsy across the lifespan. Results may empower users to make more informed choices that impact not only their lifestyle but also physical health.

## Supplementary Information

Supplementary Table S1.

## Data Availability

A subset of the data used in this analysis is available at https://www.epilepsyecosystem.org/ (Dr. Levin Kuhlmann, levin.kuhlmann@monash.edu). Under the terms of the data-sharing agreements for the patient cohorts included in this study, we are unable to share the other source data publicly.

## Code availability

All code used in this study is available at: https://github.com/WEAR-ISG/WEAR-DataQuality.
