# Supplementary material for: Data quality evaluation in wearable monitoring
Source: Sci Rep. 2022 Dec 10;12:21412. doi: 10.1038/s41598-022-25949-x (PMC9741649; doi:10.1038/s41598-022-25949-x)
Supplement: Supplementary file 1 — Supplementary Table S1. [file 41598_2022_25949_MOESM1_ESM.pdf]

Data Quality Evaluation in Wearable Monitoring

Sebastian Böttcher, Solveig Vieluf, Elisa Bruno, Boney Joseph, Nino Epitashvili, Andrea Biondi, Nicolas Zabler, Martin Glasstetter, Matthias Dümpelmann, Kristof Van Laerhoven, Mona Nasseri, Benjamin H Brinkman, Mark P Richardson, Andreas Schulze-Bonhage, Tobias Loddenkemper

Supplementary Information

Table S1: Results for statistical analysis of the comparisons. The exact p-value is omitted if it is below 0.001.

Comparison	Option	Metric	N	mean % [CI95]	p	t	dof
**Overall Signal Quality**	EDA vs. BVP	EDA	1088	70.4 [68.8,71.9]	**< 0.001**	10.57	2174
		BVP		60.2 [59,61.3]			
	EDA vs. TEMP	EDA		70.4 [68.8,71.9]	**< 0.001**	-30.43	
		TEMP		96.1 [95.4,96.8]			
	BVP vs. TEMP	BVP		60.2 [59,61.3]	**< 0.001**	-53.46	
		TEMP		96.1 [95.4,96.8]			
**Overall Study Setting**	inpatient	completeness	217	57.7 [53.9,61.5]	0.055	1.93	254
	outpatient		39	48 [38.1,57.9]			
	inpatient	on-body	217	96 [94.5,97.6]	0.996	0.00	
	outpatient		39	96 [92.8,99.2]			
	inpatient	EDA	217	74.2 [71.3,77.1]	0.057	-1.91	
	outpatient		39	81.1 [76.6,85.6]			
	inpatient	BVP	217	59.2 [56.9,61.4]	0.507	0.66	
	outpatient		39	57.2 [51.7,62.8]			
	inpatient	TEMP	217	97.9 [96.8,99]	0.553	-0.59	
	outpatient		39	98.7 [97.7,99.8]			

Comparison	Option	Metric	N	mean % [CI95]	p	t	dof
**Per-site Study Setting**	KCL inpatient	completeness	29	51.5 [41.5,61.6]	**0.011**	2.68	41
	KCL outpatient		14	30.1 [18.6,41.6]			
	MCR inpatient	completeness	19	97.9 [96.3,99.5]	**< 0.001**	4.00	31
	MCR outpatient		14	76.9 [63.8,90]			
	UKF inpatient	completeness	169	54.2 [50.1,58.3]	**0.016**	2.42	178
	UKF outpatient		11	34.1 [19.1,49]			
	KCL inpatient	EDA	29	62.7 [52.7,72.7]	**0.001**	-3.53	41
	KCL outpatient		14	88 [83.5,92.6]			
	MCR inpatient	EDA	19	78.4 [68.1,88.7]	0.717	0.37	31
	MCR outpatient		14	76 [68.5,83.5]			
	UKF inpatient	EDA	169	75.7 [72.6,78.8]	0.638	-0.47	178
	UKF outpatient		11	78.7 [66.5,90.9]			
**Overall Time of Day**	day	completeness	1080	82.8 [81.5,84.1]	**< 0.001**	-5.76	2124
	night		1046	88.5 [87.1,89.9]			
	day	on-body	1080	90.4 [89.3,91.5]	0.681	0.41	
	night		1046	90 [88.5,91.5]			
	day	EDA	1080	65.6 [64,67.2]	**< 0.001**	-7.95	
	night		1046	75.1 [73.4,76.8]			
	day	BVP	1080	49.9 [48.8,51]	**< 0.001**	-22.67	
	night		1046	70.7 [69.3,72.1]			
	day	TEMP	1080	95.5 [94.8,96.2]	0.801	-0.25	
	night		1046	95.6 [94.7,96.6]			
**BCH Device Placement**	wrist	completeness	383	98.3 [97.7,99]	0.908	-0.12	827
	ankle		446	98.4 [97.8,99]			
	wrist	on-body	383	88.2 [85.7,90.6]	0.919	-0.10	
	ankle		446	88.3 [86.3,90.4]			
	wrist	EDA	383	70.5 [68.1,73]	0.094	1.68	
	ankle		446	67.4 [64.8,70.1]			

Comparison	Option	Metric	N	mean % [CI95]	p	t	dof
	wrist	BVP	383	58 [56.1,59.9]	**< 0.001**	-3.42	
	ankle		446	62.7 [60.8,64.6]			
	wrist	TEMP	383	94.4 [92.9,95.9]	**0.015**	-2.44	
	ankle		446	96.5 [95.6,97.3]			
**Overall Recording Mode**	local	completeness	865	98 [97.5,98.5]	**< 0.001**	44.68	1086
	streaming		223	51.4 [47.8,54.9]			
